# Diabetic Nephropathy: Perspective on Extracellular Vesicles

**DOI:** 10.3389/fimmu.2020.00943

**Published:** 2020-06-03

**Authors:** Yanfang Lu, Dongwei Liu, Qi Feng, Zhangsuo Liu

**Affiliations:** ^1^Department of Nephrology, The First Affiliated Hospital of Zhengzhou University, Zhengzhou, China; ^2^Research Institute of Nephrology, Zhengzhou University, Zhengzhou, China; ^3^Key Laboratory of Precision Diagnosis and Treatment for Chronic Kidney Disease in Henan Province, Zhengzhou, China; ^4^Core Unit of National Clinical Medical Research Center of Kidney Disease, Zhengzhou, China

**Keywords:** cell–cell cross-talk, diabetic nephropathy, diagnosis biomarker, exosomes, extracellular vesicles, microvesicles

## Abstract

Diabetic nephropathy (DN) is a major microvascular complication of diabetes mellitus. It is the most frequent cause of end-stage renal disease with no definitive therapy available so far. Extracellular vesicles (EVs), including exosomes, microvesicles, and apoptotic bodies, are nano- and micron-sized heterogeneous vesicles that can be secreted by almost all cell types. Importantly, EVs contain many biologically active materials, such as RNAs, DNAs, proteins, and lipids, from their parental cells, which can be transported to their recipient cells to mediate intercellular communication and signaling. Accumulating studies demonstrated that EVs, mainly exosomes and microvesicles, participated in the pathophysiological process of DN. Recently emerging studies also found that the contents of EVs in the urine (miRNAs, mRNAs, and proteins) could be used as potential biomarkers for DN. Therefore, in this mini-review, the generation, isolation methods, and biological function of EVs were introduced, and then the current information about the mechanism and the diagnostic value in the development of DN was summarized. Moreover, the review also discussed the future challenges of exploring the role of EVs in kidney disease.

## Introduction

Extracellular vesicles (EVs) are small spherical packages released by a variety of cells into the extracellular environment. They are particularly important mediators of intercellular communication between donor cells and recipient cells (1). Exosomes and microvesicles (MVs) are two major EVs that can be distinguished from each other depending on their different sizes, expressed biomarkers (2), and different generation forms (3). Exosomes can range from 30 to 100 nm in diameter and are derived from the endocytic pathway with the invagination of the plasma membrane (4), leading to the formation of mature multivesicular bodies (MVBs). These MVBs fuse with lysosomes and the contents are degraded, or they fuse with the plasma membrane to release exosomes into the extracellular space (5–7). The microvesicles are 50–1,000 nm in size and are formed by the outward budding of the plasma membrane, with the release of a heterogeneous irregular population of large vesicles (8). The contents of both exosomes and microvesicles include various types of proteins, lipids, DNAs, and RNAs originating from their parental cells. The protein markers for exosomes belong to the family of tetraspanins, such as CD9, CD63, and CD81. The MVs contain mainly transmembrane proteins, such as integrins and selectins (9).

EVs were first proposed to be released for getting rid of cellular waste (10). However, recent studies found that EVs played an important role in maintaining cell homeostasis and were crucial in cell communication by spreading protective or injury signals to neighboring or remote cells (11). EVs can be taken up by recipient cells to induce cell–cell cross-talk by a variety of mechanisms, including endocytosis, pinocytosis, phagocytosis, and membrane fusion, or signals by directly activating cell surface receptors via ligands or presenting antigens. The uptake mechanism for a given EV may depend on proteins and glycoproteins found on the surface of both the EVs and the target cells (12). These differences reflect the heterogeneity in both EV populations and target cell types (13–15).

Diabetic nephropathy (DN) is a microvascular complication in patients with both type I and II diabetes associated with poor glycemic control. It ultimately leads to end-stage renal disease, accounting for 40% of patients requiring renal replacement therapy (16). DN is a chronic disease characterized by glomerular hypertrophy, proteinuria, decreased glomerular filtration, and renal fibrosis with loss of renal function caused by high glucose condition (17). The morphological and the ultrastructural changes in DN include progressive thickening of the glomerular basement membrane, expansion of the mesangial matrix, glomerular hyperfiltration, and tubulointerstitial fibrosis (18, 19). DN can quickly progress to end-stage kidney disease without treatment because of no clinical symptoms in its early stage. Hence, understanding the mechanism of the development of DN is extremely important for better treatment. Although microalbuminuria (MA) can be used as a biomarker of the early onset of DN, it lacks the sensitivity and the specificity to predict DN risk. In addition, the gold standard of diagnosing DN is largely based on pathological changes in renal biopsy, which is an invasive examination for patients. Several adverse events, such as infection and hemorrhage, may occur during renal puncture. Also, renal biopsy cannot be performed serially with the progression of kidney disease and is prone to sampling error. Therefore, investigating by noninvasive and sensitive biomarkers that can predict DN development is critical. Urine is a highly preferred biofluid in detecting diseases, especially kidney disease, due to its non-invasive accessibility, allowing fast and easy sampling. However, whole urine cannot be used to assess the structural changes in the kidney because most of the contents in urine come from the blood and lack content changes in the kidney itself. Since blood EVs cannot pass through the glomerular membrane, the urine EVs (uEVs) mainly come from all parts of the nephrons and collecting ducts (20, 21). Therefore, the contents of uEVs can be better candidates in diagnosing kidney diseases compared with the whole urine (22). Hence, uEVs might serve as promising cellular candidates for reliable biomarker identification besides total urine analysis. Scientists aim to find new ways to discover uEV biomarkers (including miRNAs, mRNAs, and proteins) by fully understanding the mechanisms of action of EVs in DN. Based on the latest literature reviews, this study aimed to elucidate the mechanism of action of EVs in DN and the opportunities and the challenges in discovering uEV biomarkers.

## Isolation of uEVs

Based on the different biological properties of EVs, such as density, shape, size, and surface proteins, many methods can be used for isolating uEVs, including ultracentrifugation, density gradient centrifugation, size exclusion chromatography, ultrafiltration, precipitation, and immuno-capture assays (23). Additionally, a variety of commercialized new isolation kits have emerged (24). The detailed characteristics of each isolation method have been described in many published studies and hence are not described here (25, 26). The choice of a specific isolation technique probably depends on the type of downstream analyses used for “omics” characterization (transcriptomics or proteomics). For example, in a recently published study, RNAs extracted from human urinary EVs obtained by five different urinary EV purification methods (chromatography, immune-affinity, membrane affinity, precipitation, and ultracentrifugation combined with density gradient) were analyzed by miRNA sequencing. Different methods could produce different results, including library sizes, mapping distributions, number of miRNA reads, and diversity of transcripts. EVs obtained by immune-affinity yielded the purest subset of small EVs, while purification by spin column chromatography indicated a tendency to isolate different subtypes of small EVs. Indeed different EV purification methods could isolate different subtypes of EVs with varying efficiencies, thus influencing the results of miRNA profiles (27). Isolating a pure vesicle of a subpopulation is relatively difficult because of the size overlap in the vesicle subpopulations. Differentiating between the subpopulations was difficult due to the different isolation methods and the incomplete characterization. Therefore, standard methods are urgently needed to isolate EVs from the urine.

## EV Secretion in Kidneys

EVs can be secreted by all kinds of kidney cells, such as podocytes, tubular cells (28), glomerular endothelial cells (29), and glomerular mesangial cells (GMCs) (30), under normal conditions. In the last few years, numerous studies reported that the modifications of the culture conditions could change the composition of EVs. Several examples are available about cell environments that influence the components of EVs. For example, under stress conditions such as hypoxia (31), acidic pH (32), uremic toxins (33), high glucose (34), and oxidative stress, the number of secreted EVs increases and their contents can be changed. EVs can induce the release of cytokines and promote the aggregation of inflammatory cells. In addition, EVs secreted by damaged kidney cells can be transferred to other normal kidney cells and change the phenotype of normal kidney cells to induce cell–cell cross-talk. Exosomes can be transferred from human renal proximal tubule cells to distal tubules and collecting duct cells using fluorescence labeling and co-culturing technology (28).

## EV-Mediated Intranephron Cell–Cell Cross-Talk and Signaling Pathways in DN

**Figure 1:**
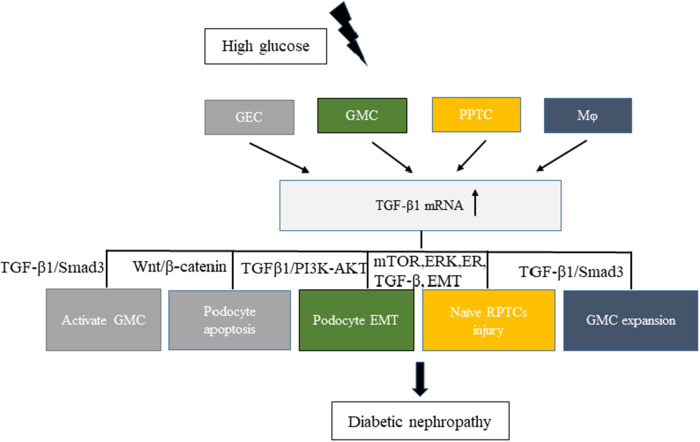
Related cell–cell cross-talk and pathways of increased levels of TGF-β1 mRNA in extracellular vesicles involved in diabetic nephropathy (DN). High glucose stimulated different cells, such as GEC, GMC, RPTC, and Mφ, to express TGF-β1 mRNA and promote DN development. Different colors in the figure represent different cell–cell cross-talks. GEC, glomerular endothelial cells; GMC, glomerular mesangial cells; Mφ, macrophages; RPTC, renal proximal tubular cells.

Hyperglycemia can cause pathophysiological changes in the kidneys because of the cellular responses to high glucose levels, including vascular cells, endothelial cells, podocytes, mesangial cells, and tubular cells. A better understanding of the molecular mechanisms underlying these cellular effects is crucial to better understand the mechanism of this disease. Increasing evidence demonstrates that high glucose stimulation can induce the abnormal secretion of EVs carrying a variety of proteins, lipids, and RNAs. Hence, EVs have emerged as a novel vector for cell–cell communication. In kidneys, the cell–cell cross-talk induced by secreted EVs on high glucose stimulation has recently been recognized to play a critical role in the development of DN and the decline in renal function (Figure 1).

The transforming growth factor-β1 (TGF-β1) is a multifunctional cytokine involved in the development of DN. It can promote glomerulosclerosis, facilitate the accumulation of extracellular matrix, enhance diabetic albuminuria, and inhibit the production and the function of mineralocorticoids in DN (35). High glucose stimulation can increase the secretion of TGF-β1 in EVs from various kidney cells. Exosomes from high-glucose-treated glomerular endothelial cells activate mesangial cells to promote renal fibrosis in DN. The increased secretion of exosomes enriched with TGF-β1 mRNA can promote α-smooth muscle actin expression, proliferation, and extracellular matrix protein overproduction in GMCs through the TGF-β1/Smad3 signaling pathway (29). Moreover, both glomerular endothelial cells and GMCs can interact with podocytes via the increased secretion of EVs enriched with TGF-β1 mRNA. Wu et al., found that exosomes from high-glucose-treated glomerular endothelial cells induced the epithelial–mesenchymal transition and the dysfunction of podocytes. Another study revealed that TGF-β1 mRNA and canonical Wnt/β-catenin signaling was involved in the exosome-induced podocyte EMT (36). The interaction between GMCs and podocytes via exosomes also affected the function of glomeruli in DN. Wang et al. (37) showed that exosomes derived from high-glucose-induced mesangial cells induced podocyte injury through the increased secretion of TGF-β and TGF-β1/PI3K-Akt signaling. Also, a cell–cell cross-talk occurs between renal proximal tubular cells (RPTCs) and naïve RPTCs. In 2019, Ravindran et al. found that, under high glucose conditions, RPTC-derived MVs activated key signaling pathways (mTOR, ERK, ER, TGF-β, and EMT) in naïve RPTCs, thus inducing renal cell injury and DN development (38). Macrophages and glomerular endothelial cells induced the fibrosis of GMCs through the TGF-β1/Smad3 pathway. In a study published in 2019, Zhu et al., found that, under high glucose conditions, macrophage-derived exosomes induced mesangial cell proliferation, activation, and overproduction of fibrotic and inflammatory factors both *in vivo* and *in vitro*. Another study showed that exosomes with TGF-β1 mRNA might be the key factor promoting mesangial expansion and renal fibrosis, thus providing new insights into the pathologic mechanism of DN (39).

EV contents affect the development of DN. Glomerular endothelium dysfunction, which plays a crucial role in the pathogenesis of early DN, was affected by plasma platelet microparticles in rats with early DN. A recent study by Zhang et al. (40) found that platelet microparticles with the increased expression of CXCL7, which activated the mTORC1 pathway, mediated glomerular endothelial injury in rats with streptozotocin-induced diabetes and early DN. The uEVs of patients with type 2 diabetes expressed high levels of miR-15b-5p, leading to the apoptosis of mouse mesangial cells through decreasing the translation of Bcl-2 (41). Hence, miR-15-5p promoted DN.

## uEVs in the Diagnosis of DN

EVs can be found and isolated in several body fluids such as plasma (42), urine (43), amniotic fluid (44), saliva (44), semen (2), and so on. The lipid bilayer of EVs can protect proteins from proteolytic cleavage and genetic information from degradation during transiting into the extracellular environment (45, 46). Most importantly, EVs are stable during long-term storage and repeated freeze–thaw cycles (47–49). The number and contents of EVs in the urine could be changed during high-glucose-induced kidney injury. With the rapid development of second-generation sequencing and mass spectrometry technologies, detecting EVs using the combined proteomics, lipidomics, metabolomics, and comprehensive genomic analyses, together with advanced systems biology algorithms, may facilitate major new discoveries benefiting patients with DN. Table 1 shows a list of recently published studies on the role of uEVs (including miRNAs, mRNAs, and proteins) in diagnosing DN.

**Table 1:** Examples of EV biomarkers in diagnosis DN.

**Biomarkers**	**DN type**	**Marker**	**Source/origion**	**Isolation method**	**References**
miRNAs	Type 2 DN	Combined let-7i-5p, miR-15b-5p, miR-24-3p, and miR-27b-3p	Urinary extracellular vesicles	miRCURY™	(50)
				Exosome Isolation Kits	
	Type 2 DN	miR-362-3p, miR-877-3p, miR-150-5p, and miR-15a-5p	Urinary exosomes	Ultracentrifuge	(51)
	Type 2 early DN	miR-192	Urinary extracellular vesicle	Ultracentrifuge	(52)
	Type 1 DN	miR-130a,miR-145, miR-155, and miR-424	Urinary exosomes	Ultracentrifuge	(53)
	Type 2 DN	miR-320c	Urinary exosomes	Exosome precipitation reagent ExoQuick-TC	(54)
	DN rats	miR-451-5p	Urinary exosomes	Differential centrifugation	(55)
mRNAs	Type 1 and 2 DN	WT1 mRNA	Urinary exosomes	Ultracentrifuge	(56)
Proteins	Type 1 and 2 DN	Regucalcin	Urinary exosomes	Combination of an ultracentrifugation-based protocol with DTT treatment of the low-speed pell	(57)
	Type 1DN	WT1 protein	Urinary exosomes	Differential centrifugation method	(58)
	Type 1 and 2 DN	AMBP, MLL3, and VDAC1 protein	Urinary exosomes	Combination of an ultracentrifugation-based protocol with DTT treatment of the low-speed pell	(59)
	Type1 DN	Cystatin B and NGAL	Urinary exosomes	Hydrostatic filtration dialysis	(60)

miRNAs are small noncoding RNAs with 18–22 nucleotides. They are involved in a variety of cellular processes. They can be found in the extracellular environment and the urine. Hence, miRNAs can function as paracrine or endocrine signals between cells. Some of these circulating miRNAs are encapsulated in various EVs (61). Specific circulating miRNAs can act as noninvasive diagnostic biomarkers for a wide variety of diseases and conditions (51, 62). In 2016, a study by Jia et al., on 80 patients with type 2 diabetes and normoalbuminuria (*n* = 30), MA (*n* = 30), or macroalbuminuria (*n* = 20), besides 10 healthy controls, found that miR-192 in urinary EVs acted as a biomarker of DN. The miR-192 levels increased in early DN in the microalbuminuric group but decreased in the normoalbuminuric, control, and macroalbuminuric groups. uEV miR-192 might be a noninvasive tool for detecting early DN in patients with type 2 diabetes (52). Prabu et al. (50) also found that a combination of let-7i-5p, miR-15b-5p, miR-24-3p, and miR-27b-3p in urinary microvesicles could be used to diagnose patients with type 2 DN having an area under the curve (AUC) of 0.867. A previous study found that miR-145 levels increased not only in patients with type 1 diabetes and incipient DN but also in animal models of early experimental DN and mesangial cells with high glucose treatment, indicating that miR-145 might serve as a candidate biomarker in diagnosing DN (53). In 2016, Deli et al. found that miRNA-320c, which was indirectly involved in TGF-β signaling via targeting TSP-1, might represent a novel candidate marker for the early progression of type 2 DN (54). In the same year, Mohan et al. (55) also found that miR-451-5p, identified using small RNA sequencing and qPCR in urine exosomes, might be an early candidate biomarker of DN in rats. Studies on internal references in uEVs also emerged. A previous study demonstrated that HY3 and RNU48 might serve as optimal endogenous controls for uEV miRNA expression analysis (63). This highlighted the importance of EV-derived miRNAs in the diagnosis of DN.

mRNAs in uEVs can also be used as biomarkers in the diagnosis of DN. Wilm's tumor-1 (WT1) plays an important role in the homeostasis of mature podocytes. It can regulate many genes in mature podocytes, including podocalyxin (64), nephrin (65), BMP7, and so on (66). A recent study by Hideharu et al., which involved 20 patients with overt or heavy proteinuria due to DN or minimal-change nephrotic syndrome and five healthy controls, found that WT1 mRNA in urine exosomes could be used as a biomarker in the diagnosis of type 1 DN with the AUC of 0.705 (56).

The major trait of DN is proteinuria. The proteins are often abundant in whole urine, making the detection of low-abundance proteins difficult. However, the exosomal proteome implies a reduction in complexity compared with whole urine, with a much lower dynamic range of protein concentrations and therefore a better chance to detect low-abundance proteins as candidate biomarkers. With the development of proteomic techniques, a large number of studies on the biomarkers of proteins in EVs for the diagnosis of DN emerged in recent years (67). For example, a study published in 2015 screened 34 different proteases and 32 protease inhibitors, revealing that proteases and protease inhibitors in uEVs isolated by hydrostatic dialysis might serve as predictors of DN. The levels of myeloblastin and its natural inhibitor elafin increased in the normoalbuminuric and the microalbuminuric groups. The study also found that the levels of cystatin B, a natural inhibitor of cathepsins L, H, and B as well as of neutrophil gelatinase-associated lipocalin, increased in the normoalbuminuric group, indicating that the aforementioned proteases and protease inhibitors might serve as diagnostic markers of DN (60). In 2014, another study conducted by Zubiri et al. found the levels of a panel of three proteins (AMBP, MLL3, and VDAC1) in urinary exosomes were changed by using nano-liquid chromatography-tandem mass spectrometry, which suggested they were promising noninvasive candidates to detect DN (59). Apart from this, they also found that urinary exosomal regucalcin declined in DN patients (57). In addition, the expression of WT1 protein in urinary exosomes from spot urine samples of patients with type 1 diabetes mellitus (*n* = 48) and healthy controls (*n* = 25) was analyzed. The findings showed that the levels of urinary exosomal WT1 protein were significantly higher (*P* = 0.001) in patients with proteinuria than in those without proteinuria, suggesting that WT1 could be used as an early noninvasive marker of DN (58).

## Discussion

EVs play important roles in DN onset and development. However, previous studies focused only on exosomes or MVs instead of a mixture of EVs, hindering the detailed exploration of the physiological and the pathological processes of DN. Moreover, nearly all results describing the EV-mediated cell–cell cross-talk were based on experimental data *in vitro* or partly on rodent models. Hence, further studies were needed to extend these findings.

EVs can also be used as biomarkers in the diagnosis of DN because of their various functions in kidney disease progression. However, the discovery of markers has many challenges. First, the low purity of uEVs obtained by different isolation methods is common in studies on uEV biomarkers. Second, most of the studies focused on mRNAs, miRNAs, and proteins in the discovery of DN biomarkers. Few studies investigated other contents in the urine, such as lncRNAs, circRNAs, and DNA. Further, the function of EV miRNAs in DN is less explored. Third, the number of patients in previous studies on discovering the biomarkers of DN was small. Hence, validation studies with large cohorts are required for biomarker development and clinical translation. In conclusion, EVs play an important role in DN and hence can be used as a biomarker of DN. Further in-depth studies should be conducted on the roles of EVs in the development of DN, thus providing a theoretical basis and additional intervention targets for DN therapy.

## Author Contributions

ZL conceptualized the ideas. YL performed the literature search, drafted the original manuscript, and drew the figures. DL and QF revised the manuscript. All the authors approved the final version of the manuscript.

## Conflict of Interest

The authors declare that the research was conducted in the absence of any commercial or financial relationships that could be construed as a potential conflict of interest.
